# Capsicumicine, a New Bioinspired Peptide from Red Peppers Prevents Staphylococcal Biofilm *In Vitro* and *In Vivo* via a Matrix Anti-Assembly Mechanism of Action

**DOI:** 10.1128/Spectrum.00471-21

**Published:** 2021-10-27

**Authors:** Rafael Gomes Von Borowski, Sophie Chat, Rafael Schneider, Sylvie Nonin-Lecomte, Serge Bouaziz, Emmanuel Giudice, Aline Rigon Zimmer, Simone Cristina Baggio Gnoatto, Alexandre José Macedo, Reynald Gillet

**Affiliations:** a Université de Rennes, CNRS, Institut de Génétique et de Développement de Rennes (IGDR), UMR6290, Rennes, France; b Programa de Pós-Graduação em Ciências Farmacêuticas, Faculdade de Farmácia, Universidade Federal do Rio Grande do Sul, Porto Alegre, Brazil; c Université de Paris, CNRS, CiTCoM (Cibles Thérapeutiques et Conception de Médicaments) UMR 8038, Faculté de Pharmacie, Paris, France; d Centro de Biotecnologia da Universidade Federal do Rio Grande do Sul, Porto Alegre, Brazil; INTHERES

**Keywords:** antibiofilm, biofilm, matrix, peptides, anti-assembly, resistance, tolerance, *Staphylococcus*, antimicrobial peptides

## Abstract

Staphylococci are pathogenic biofilm-forming bacteria and a source of multidrug resistance and/or tolerance causing a broad spectrum of infections. These bacteria are enclosed in a matrix that allows them to colonize medical devices, such as catheters and tissues, and that protects against antibiotics and immune systems. Advances in antibiofilm strategies for targeting this matrix are therefore extremely relevant. Here, we describe the development of the *Capsicum* pepper bioinspired peptide “capsicumicine.” By using microbiological, microscopic, and nuclear magnetic resonance (NMR) approaches, we demonstrate that capsicumicine strongly prevents methicillin-resistant Staphylococcus epidermidis biofilm via an extracellular “matrix anti-assembly” mechanism of action. The results were confirmed *in vivo* in a translational preclinical model that mimics medical device-related infection. Since capsicumicine is not cytotoxic, it is a promising candidate for complementary treatment of infectious diseases.

**IMPORTANCE** Pathogenic biofilms are a global health care concern, as they can cause extensive antibiotic resistance, morbidity, mortality, and thereby substantial economic loss. So far, no effective treatments targeting the bacteria in biofilms have been developed. Plants are constantly attacked by a wide range of pathogens and have protective factors, such as peptides, to defend themselves. These peptides are common components in Capsicum baccatum (red pepper). Here, we provide insights into an antibiofilm strategy based on the development of capsicumicine, a natural peptide that strongly controls biofilm formation by Staphylococcus epidermidis, the most prevalent pathogen in device-related infections.

## INTRODUCTION

Antimicrobial failure is a worldwide challenge, currently addressed by a WHO global action plan (1). A lack of new antibiotics and the inappropriate use of older treatments mean that multidrug-resistant strains are increasing (2). This process is favored by biofilm development, and microorganisms enclosed in biofilm matrices have antibiotic resistance that is up to 1,000 times higher than that of planktonic ones (3). This makes the matrix itself an important target for biofilm control. Biofilms are organized microbial clusters made of a self-assembled matrix that usually attaches to a surface, whether abiotic (medical devices such as catheters, teeth, etc.) or biotic (host tissues, mucus, chronic wounds, etc.) (4, 5). Since the bacteria are embedded into this matrix, they are harder to treat because of their increased tolerance and resistance to antibiotics, disinfectants, and host defenses (6, 7). Other advantages over planktonic forms include physiological and biochemical changes, beneficial quorum sensing, higher (up to 100 times) mutation rates, and persister cell development (8–10). Staphylococci are the most frequent sources of nosocomial infections, particularly Staphylococcus epidermidis and Staphylococcus aureus. While S. aureus expresses many virulence factors such as toxins and proteases, for S. epidermidis, the formation of biofilm is the most important mechanism in infection development (11). Staphylococcus epidermidis is the most frequent coagulase-negative staphylococcal (CoNS) infection-causing disease (12), surviving on various surfaces for months (13). It is present in 30% of health care-involved bloodstream infections and is significantly associated with medical device infections, including 15 to 40% of prosthetic valve endocarditis (14) and 30 to 43% of prosthetic orthopedic device infections (15). More than 150 million intravascular catheters are used per year in the United States, and there are about 250,000 catheter-related infections (16, 17). These bacteria are developing antibiotic multiresistances, such as elevated glycopeptide MICs (18), and 73 to 88% of isolates display resistance to oxacillin, fluoroquinolones, macrolides, clindamycin, and trimethoprim-sulfamethoxazole (19–21).

In this circumstance, the extracellular matrix is a complex physicochemical barrier representing one of the biggest challenges in microbial treatments (4). Therefore, the development of antivirulence strategies, such as antibiofilm agents, is crucial for the current antibiotic crisis, and peptides are an increasing arsenal for controlling pathogenic biofilms (22–24). Here, we describe the discovery of the antibiofilm peptide capsicumicine, inspired by natural peptides from the seeds of the red pepper *Capsicum baccatum.* Our data suggest that capsicumicine is a nonantibiotic peptide displaying a matrix anti-assembly (MAA) mechanism of action by interfering with polysaccharides. Notably, we report an *in vivo* anti-infective proof of concept toward the use of capsicumicine for complementary treatment of infectious diseases.

## RESULTS

### Capsicumicine prevents biofilm formation without antibiotic activity.

**FIG 1:**
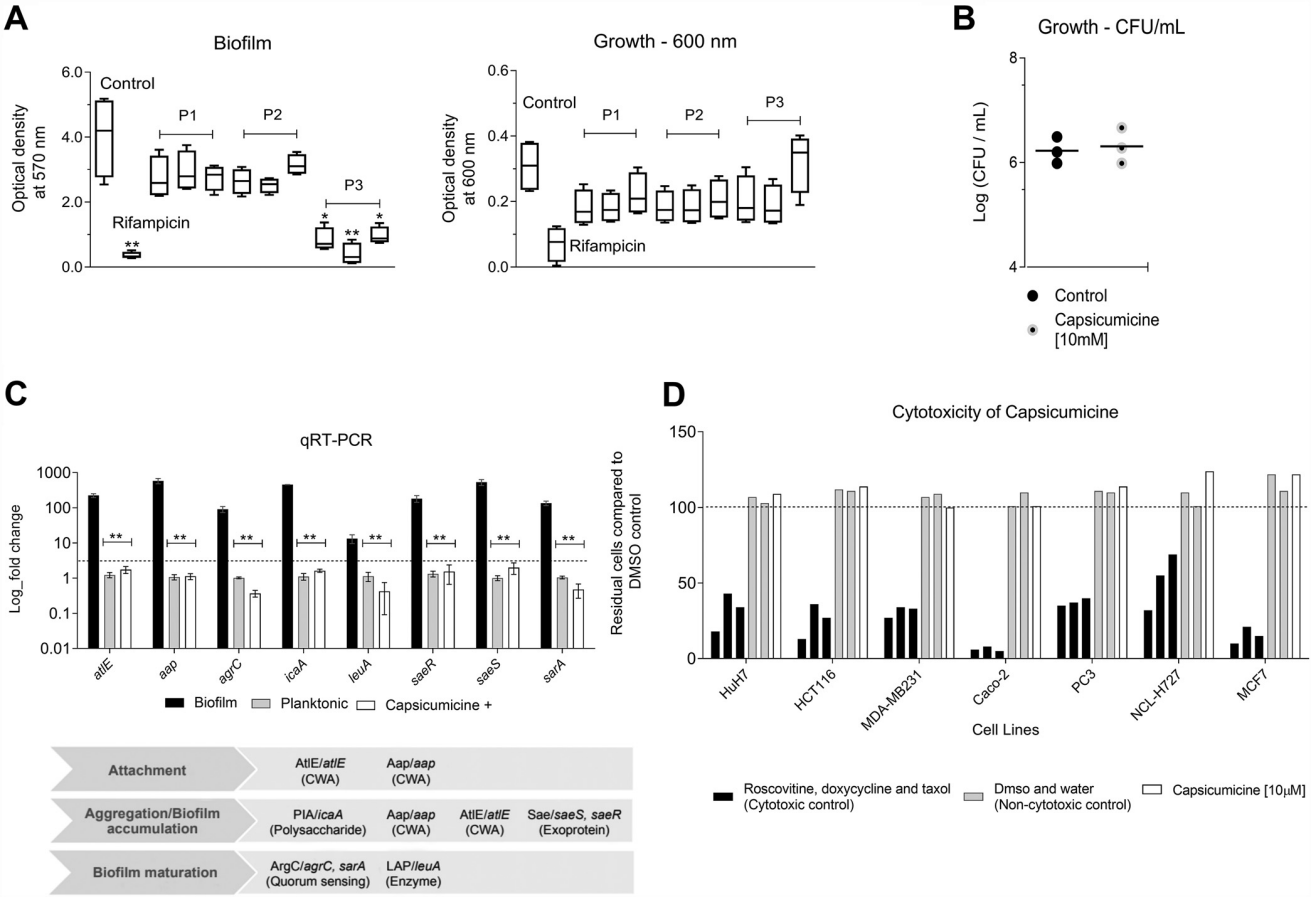
Antibiofilm activity of bioinspired peptides and cytotoxicity. (A) Antibiofilm activity of peptides P1, P2, and P3 (capsicumicine) at 1, 10, and 100 μM. Quantification of Staphylococcus epidermidis (ATCC 35984) biofilm and growth were done at an optical density of 570 (crystal violet) and 600 nm (without crystal violet), respectively, bacteria without peptide exposure (control) and the antibiotic control, 16 μg/ml rifampin. Student’s *t* test, *, *P *≤ 0.05; **, *P *≤ 0.01. (B) Growth of bacteria at 10 μM using CFU. (C) Gene expression (mean log fold changes ± standard errors of the mean) of the encoding genes involved in S. epidermidis biofilm formation compared to the planktonic (gray) and biofilm controls (black) with the *ssrA* gene used as a reference. The group exposed to capsicumicine is shown in white. CWA, cell wall-anchored proteins. Two-way analysis of variance (ANOVA) and Tukey's multiple-comparison test, **, *P* ≤ 0.0015. (D) Capsicumicine cytotoxicity evaluation in representative human cell lines shown via automated image-based cellular content analysis. HuH7, hepatocellular carcinoma; Caco-2, colorectal adenocarcinoma; MDA-MB231, breast adenocarcinoma; HCT116, colorectal carcinoma; PC3, prostatic adenocarcinoma; NCL-H727, lung carcinoma; MCF7, breast cancer. Cell counts are presented as residual cell percentages (%) compared to the average of the DMSO control, with water control also shown (gray bars). The black bars on the left show cytotoxic controls (roscovitine, doxycycline, and taxol), while the white bars are cells exposed to 10 μM capsicumicine.

We synthesized the following three peptides inspired by a natural antibiofilm fraction previously identified from *Capsicum baccatum* var. *pendulum* pepper seeds (25): P1 (RVQSEEGEDQISQRE), P2 (RAEAFQTAQALPGLCRI), and P3 (RSCQQQIQQAQQLSSCQQYLKQ). To find the most active one, we exposed these compounds to strong biofilm-forming S. epidermidis RP62A (ATCC 35984). After 24 h, crystal violet was used to quantify the remaining biofilm (Fig. 1A). P3, named “capsicumicine,” was the most active with particularly strong antibiofilm activity. Biofilm decreases were observed at all tested concentrations but especially at 10 μM. There, biofilm was reduced by over 91%, independently of cell growth inhibition (Fig. 1B). To examine the effects of capsicumicine on growth, we checked S. epidermidis CFU counts after peptide exposure. As expected, the CFU were unchanged by capsicumicine, so the peptide’s biofilm inhibition is not due to bactericidal activity (Fig. 1B). To verify the interactions between capsicumicine and established biofilm, we exposed a preexisting S. epidermidis biofilm to a single concentration (100 μM) of the peptide. At that concentration, capsicumicine accounts for 15% of the disruption of preexisting biofilm (see Fig. S1 in the supplemental material). To study the peptide’s possible mechanisms of action, we selected several genes involved in different stages of biofilm development (*atlE*, *aap*, *agrC*, *icaA*, *leuA*, *saeR*, *saeS*, and *sarA*); primers are listed in Table S1 in the supplemental material. Fold changes were analyzed by quantitative real-time PCR (qRT-PCR). Since exposed bacteria remain planktonic, we compared their relative gene expressions to planktonic control cells. For all tested genes, capsicumicine-exposed cells show the same fold changes as the control (Fig. 1C).

### Capsicumicine is not cytotoxic in mammalian cells.

To ensure that capsicumicine is safe before propose *in vivo* trials, we verified its cytotoxicity in seven different representative human cell lines. We used automated image-based cellular content analysis and found that capsicumicine-treated cells perform exactly the same as untreated controls, displaying no cytotoxicity (Fig. 1D).

### Independently of cell interactions, capsicumicine impairs biofilm attachment, aggregation, and accumulation.

**FIG 2:**
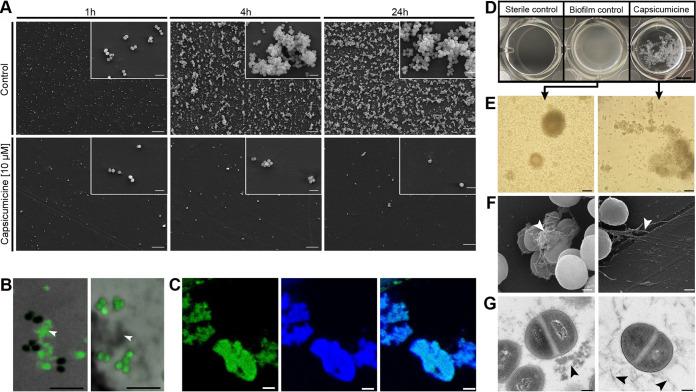
Biological characterization of capsicumicine by microscopic approaches. (A) SEM images of polystyrene coupons after 1, 4, or 24 h of culture with Staphylococcus epidermidis (ATCC 35984). (Top) Peptide-less biofilm control. (Bottom) Cultures exposed to 10 μM capsicumicine. Magnification ×500, with insets at ×5,000; scale bars, 10 μm. (B) CFM images. (Left) S. epidermidis after exposure to 10 μM capsicumicine-FITC with the peptide in green (fluorescence) and the bacterial cells in black (no fluorescence). (Right) Control, S. epidermidis after exposition to pseudonajide-FITC, an antibacterial peptide, this time showing the extracellular components in gray (no fluorescence). White arrows indicate extracellular content. Scale bars, 5 μm. (C) CFM images. S. epidermidis after exposure to 10 μM capsicumicine-FITC (left) and after exposure to concanavalin A conjugates (selectively reveals saccharides) (center) and their colocalization (right). Scale bars, 5 μm. (D to G) The organizational state of S. epidermidis biofilm after 24 h in the presence (right) or absence (control, left) of capsicumicine. (D) Macroscopic examination by pictures from the bottom of 24-well plates. The “sterile control” shows no bacteria or biofilm formation, the “biofilm control” shows homogenous adhered layers of bacteria, and “capsicumicine” shows nonadhered bacteria but agglutinates. Scale bar, 5 mm. (E) TLM images show the biofilm control with overlapping attached bacterial clusters, while the capsicumicine-exposed culture shows agglutinated nonadhered cells. Scale bar, 20 μm. (F) SEM images show the control with dense globular-like matrix features, while the capsicumicine-exposed culture shows fibrillary branch-like oligomer structures. Scale bar, 200 nm. (G) TEM images of the biofilm control show denser assembled structures, while the capsicumicine-exposed displays thin fibrillary oligomer structures. Scale bar, 200 nm. Arrows indicate the matrices.

To explore its activity during the first stages of biofilm development, we analyzed biofilm cultures on polystyrene coupons with and without capsicumicine after 1, 4, and 24 h. Scanning electron microscopy (SEM) analysis shows that bacterial attachment decreases after 1 h of capsicumicine exposure, with biofilm accumulation and cell aggregation profiles strongly reduced after 4 and 24 h (Fig. 2A). This demonstrates that capsicumicine prevents S. epidermidis coupon adhesion, and notably this activity still occurs after 24 h of incubation. In order to localize the peptide, we used capsicumicine conjugated to fluorescein isothiocyanate (capsicumicine-FITC) and confocal fluorescence microscopy (CFM). Analysis of the CFM images showed that capsicumicine-FITC stays associated with extracellular components, not entering into bacterial cells or the walls or membranes (Fig. 2B).

### Capsicumicine disturbs S. epidermidis matrix assembly.

Since capsicumicine’s antibiofilm activity was not associated with direct bacterial interactions or gene expression modulation, we used various microscopic approaches to investigate the interactions between the peptide and the extracellular matrix. In the biofilm control, macroscopic observation shows a homogenous whitish adhered layer covering the walls and bottoms of the well (Fig. 2D, middle), but when capsicumicine is present, we see whitish flocculent nonadhered heterogeneous agglutinates (Fig. 2D, right). Transmitted light microscopy (TLM) images match with these macroscopic observations (Fig. 2E). Crucially, SEM and transmission electron microscopy (TEM) techniques yield ultrastructural descriptions that support these results, with the control biofilm matrix showing denser assembled globular-like structures, while the capsicumicine-exposed matrix is less dense and has thin fibrillary branch-like structures (Fig. 2F and G). However, cellular morphologies are not different from the controls. Therefore, different imaging techniques prove that matrix assembly changes when capsicumicine is present.

### Capsicumicine interacts with matrix polysaccharides.

To explore the peptide’s affinity with these major matrix components, we exposed S. epidermidis cultures to capsicumicine (-FITC; green) and saccharide staining, and then analyzed them all by CFM. We used concanavalin A and calcofluor to selectively target matrix saccharides (blue). Amounts of capsicumicine-FITC appear exclusively on the matrix (Fig. 2B left; see also Fig. S2B in the supplemental material), and its colocalization indicates the interaction between both marked elements (Fig. 2C, right; see also Fig. S2A). Visualization was done by individual (Fig. 2C, left and center) and colocalization fluorescence (Fig. 2C, right). The peptide control was pseudonajide-FITC, an antibacterial peptide; it showed green fluorescence in the cells but not in the matrix (Fig. 2B right; see also Fig. S2C). To explore whether capsicumicine features are compatible with carbohydrate-binding modules (CBMs), we performed a BLAST and amino acid alignments between capsicumicine and chitin-, chitosan-, and polysaccharide intercellular adhesin (PIA)-binding proteins (see Table S2 in the supplemental material). UniProt tools (26) and CAZy information crossing (27) showed that capsicumicine does in fact present CBM homology with all tested proteins (see Fig. S3 in the supplemental material).

### Capsicumicine shifts staphylococcal synthetic matrix.

**FIG 3:**
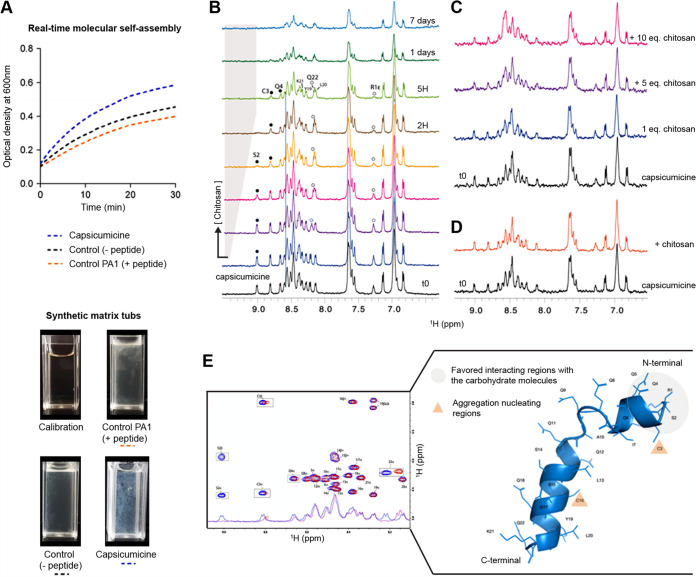
Molecular interactions between capsicumicine and target-saccharides. (A) RTMSA curves of artificial staphylococcal biofilm assembly. Optical densities (OD_600_) were recorded as a function of the time in the presence of capsicumicine (blue dots) or PA1 as peptide control (orange dots) and the synthetic matrix without peptides (black dots); each reaction tub is shown under the graph. (B-D) NMR titration of capsicumicine by chitosan at 600 MHz, 10°C. (B) From the bottom to the top: increasing concentrations of soluble chitosan [<0.5 eq. and 8% of volume variation] were gradually added to capsicumicine water solution. Specific line broadenings and frequency shifts are shown in the NMR spectrum for some amide protons upon titration; white bullets for terminal R1 and Q22 and black bullets for S2, C3 and Q4. (C) Assembled chitosan gradually added to soluble capsicumicine [0.5 eq.]; an overall line broadening was observed. (D) Assembled chitosan suddenly added to soluble capsicumicine [0.5 eq.]; the orange spectrum was recorded after few minutes of stirring. (E) Superposition of capsicumicine 1D NMR and TOCSY spectra immediately after sample preparation (blue) and chitosan [10 eq.] addition (red) and; capsicumicine structure predicted by Phyre2 server; according to NMR results the aggregation nucleating regions are shown as orange triangles and favored interacting region with sugar moieties is shown in gray.

**FIG 4:**
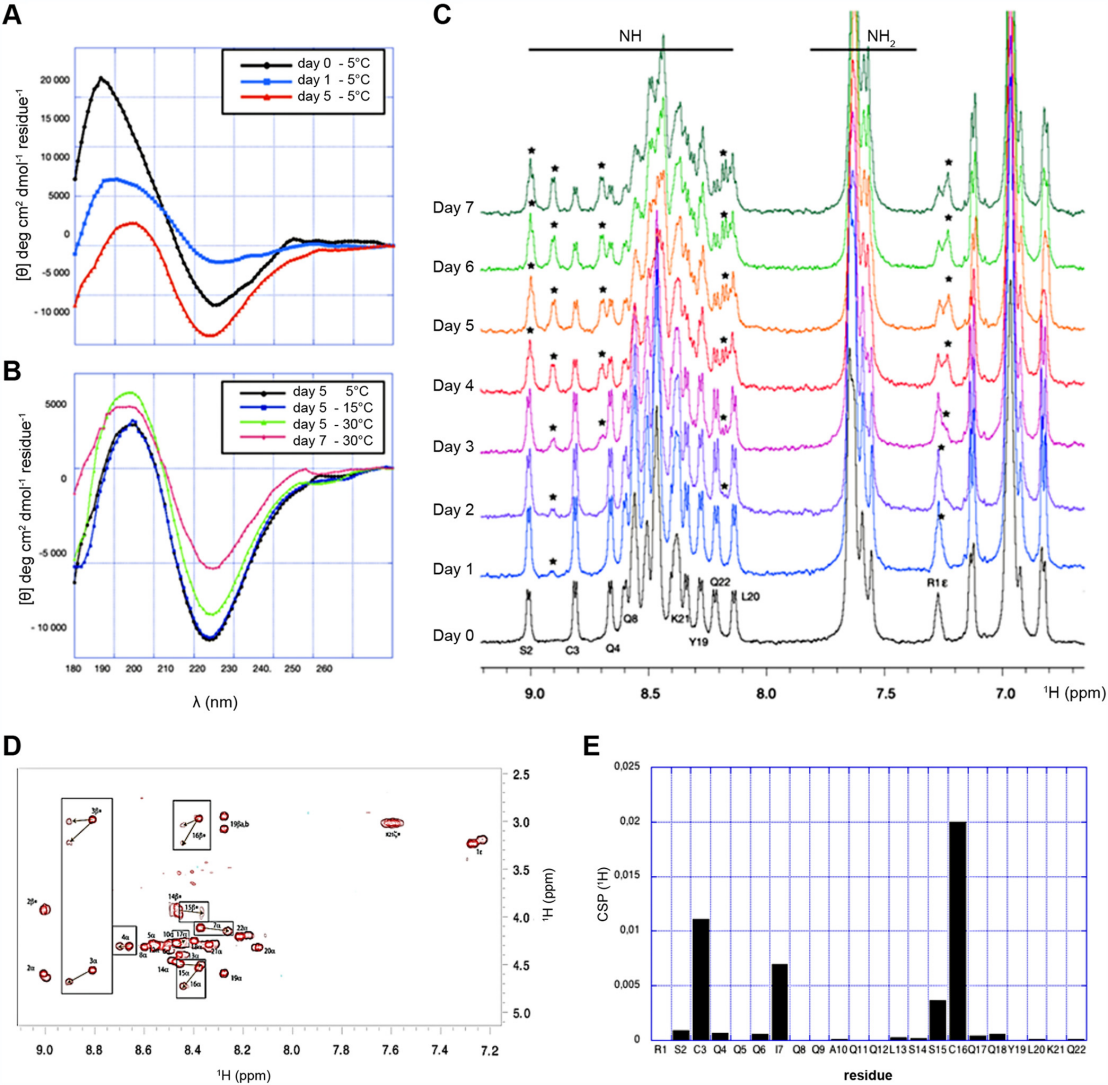
Capsicumicine conformational changes. CD spectra. (A) Evolution at 5°C over 5 days. (B) Evolution versus time and temperature. (C) NMR spectra recorded at pH 5.0, 0.3 mM, and 10°C immediately after extemporaneous preparation (bottom); a second conformation appears over the time corresponding to the second set of amide resonances (stars). (D) Superposition of capsicumicine TOCSY spectra immediately after sample preparation (red) and at day 5 (brown). The most important chemical shift perturbations (CSP) between the original and the new conformations are boxed and clustered around the two cysteines. (E) Proton chemical shift perturbations computed from the TOSCY spectra are displayed in the box.

To confirm these interactions in the absence of bacteria metabolic or regulatory influences, we adapted a model of artificial staphylococcal biofilm assembly to test it. For this purpose, we used chitosan in order to mimic natural poly-*N*-acetylglucosamine (PNAG) biofilm. Briefly, we monitored the real-time molecular self-assembly (RTMSA) reaction (based on the assembly reaction of the synthetic staphylococcal matrix; see Materials and Methods) by measuring the optical density at 600 nm (OD_600_) as a function of time with or without capsicumicine. OD increases when capsicumicine is present, which shows that the molecular self-assembly reaction is quicker overall (Fig. 3A). The profiles of assembled matrices are visually different, with larger agglutinates in the presence of capsicumicine, although both controls are similar (Fig. 3A). Remarkably, these profiles are comparable to those previously observed in the presence of bacteria (Fig. 2D). To evidence the interactions between capsicumicine and target saccharides, we performed nuclear magnetic resonance (NMR) titration experiments using chitosan as a mimic of the matrix PNAG. The evolutions of the capsicumicine NMR spectra upon chitosan addition were monitored. Reference one-dimensional (1D) proton NMR spectra were recorded at all tested conditions. Chitosan was gradually added from a concentrated solution, and spectra changes were monitored. This solution remained clear during the 7 days of recording. The addition of soluble chitosan drives noticeable changes of the resonance frequency of some of the amide protons mainly in the N-terminal part of the peptide (S2, C3, Q4, and R1 [not shown]) (Fig. 3B). Conversely, to Y19, L20, and K21 NH and the Y19 aromatic proton resonances, between 6.8 and 7.2 are not sensitive to the presence of chitosan (Fig. 3E). The concentration variation of chitosan was estimated to be less than 0.5 eq at the end of the titration. This small quantity of chitosan drives spectral modifications in the N-terminal part of capsicumicine and strongly supports the interaction between both partners. The important broadening observed after 1 day shows that the peptide structure keeps evolving over time and that this is not due to cysteine oxidation (Fig. 4D and E). These experiments were repeated, adding gelled chitosan instead of soluble chitosan, mimicking an assembled matrix. It does not drive any significant spectral changes except for a general line broadening, which probably arises from an increased viscosity of the solution (Fig. 3C). The sudden addition of gelled chitosan pellets in the NMR solution does not modify the spectrum either (Fig. 3D).

### Structural requirements toward molecular interactions with target saccharides.

To better understand the mechanisms involved in capsicumicine bioactivity, we step toward the knowledge of its conformation in solution and molecular interactions with matrix representative saccharides. To monitor the conformation of free capsicumicine in response to time and temperature, we first used circular dichroism (CD) and nuclear magnetic resonance (NMR) spectroscopies. Spectral deconvolution using the CDSSTR algorithm discloses a helical structure (about 43 to 44%) with a nonnegligible proportion of β strands (31 to 33%) and unfolded conformations (about 20%) (see Table S3 in the supplemental material). The structure stability was assessed over 5 days, recording the CD spectra at 5°C (Fig. 4A). The main structure observed over the first 24 h is helical. After 5 days, deconvolutions show a slight decrease in the helical content. Furthermore, on the fifth day, CD spectra were also recorded at 15 and 30°C (Fig. 4B). The helical content decreases to 18 to 23%, while the β strand and turn mean proportions rose around 37% and 23%, respectively. The proportion of unfolded structures also increased. After 2 days at room temperature, a new CD spectrum was recorded at 30°C. The helix proportion became less than 15% in favor of β strand and unfolded conformations (respectively, 38 to 41% and 31 to 35%). During these days, the peptide solution remained clear, showing no macroscopic signs of aggregation. The 1D and two-dimensional (2D) NMR spectra of a 0.3 mM freshly prepared solution of capsicumicine were recorded at 10°C, pH 5.0. All amide resonances were unambiguously assigned using total correlation spectroscopy (TOCSY) and nuclear Overhauser effect spectroscopy (NOESY) spectra, except for Q9 and Q11 residues (see Fig. S4 in the supplemental material). The spectral dispersion and the spreading of the amide proton resonances disclose that the peptide is folded with one conformation in these conditions. In accordance with CD spectra, a second set of NH resonances appears as a function of time (Fig. 4C and D), revealing conformational changes in the slow exchange regime on the NMR time scale. After 5 days, the most important chemical shift perturbations (CSP) observed on the TOCSY spectrum are clustered around the two cysteines apart from the two terminal residues (boxed cross-peaks) (Fig. 4E). Interestingly, I7 located one helix turn apart from C3 is among the most sensitive residues to the conformational perturbation. This is due to a disulfide bridge (DB), since no reducing agent was added. Consequently, large proton chemical shift perturbations would be expected for every −NH amino acid, and this was not detected (Fig. 4D and E). The prevalence of the second conformation reaches more than 50% after 7 days (Fig. 4C, top). The solution remained clear over all of the spectra recording time.

### Capsicumicine attenuates the dynamics of S. aureus (Xen36) infection on a central venous catheter.

**FIG 5:**
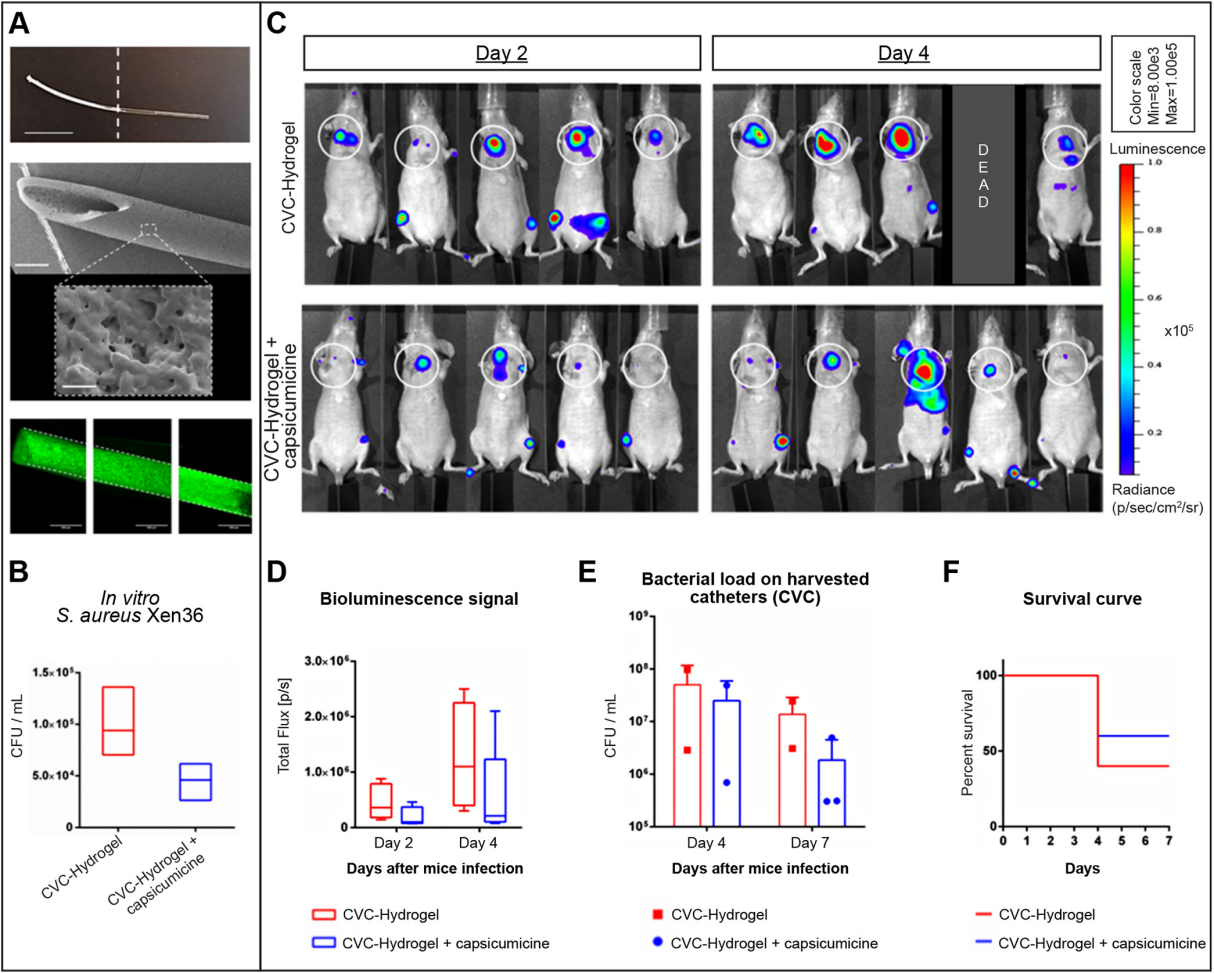
Assessment of capsicumicine precoated central venous catheter (CVC) in mice (SKH1) infected by Staphylococcus aureus Xen36. (A and B) *In vitro* CVC development. (A) From the top to the bottom, a picture of up to half capsicumicine precoated CVC (whitish) (scale bar, 1 cm), SEM image of precoated CVC (scale bar, 0.5 mm and its magnification scale bar, 1 μm), and a compilation of images of fluorescence microscopy of precoated CVC (capsicumicine-FITC in green). (B) Bacterial load on CVC. (C to F) *In vivo* CVC assessment. (C) IVIS images (radiance thresholded and smoothed with a bidimensional Gaussian filter) of ventral position for each mouse at each time point. The control group (CVC-hydrogel) is shown in the top and the treated (CVC-hydrogel + capsicumicine) in the bottom. White circles delimitate the CVC localization, and the luminescence scale is presented in radiance. (D) Bioluminescence quantification (ROI mean signals ± standard deviation [SD]) is shown as photons total flux (p/s) for each group at each time point. (E) Bacterial load (CFU/ml) on harvested CVC for each mouse at each time point (day 4 and 7 after infection). (F) The Kaplan-Meier survival curve (% of survival) at each group during the 7 days after infection. All bioluminescence data and analyses were performed using IVIS Spectrum of Perkin Elmer. All experiments were conducted in accordance with ethical committee and the French authorities (*n* = 5/group). Bioluminescence images are presented with a defined pseudocolor scale to visualize the intensity of signals emitted.

We performed a translational proof of concept evaluating long-term bacterial biofilm and related infection in mice implanted with capsicumicine precoated central venous catheter (CVC). These CVCs were previously coated using an immobilization polymer (“hydrogel”) encompassing capsicumicine. This coating confers amorphous and biocompatible surfaces to CVC (Fig. 5A). They were first validated *in vitro*, decreasing ≥51% of S. aureus colonization (Fig. 5B). Then, bacterial development was evaluated *in vivo* by bioluminescence imaging and bacterial load of harvested CVC (Fig. 5C to F). Two days (D2) after S. aureus systemic infection, 40% of the control group (CVC-hydrogel) presented a high bioluminescence signal (red zones) related to region of interest (ROI) against none in the treated group (CVC-hydrogel + capsicumicine) (Fig. 5C). Four days (D4) after infection, 75% of the control presented high ROI red zones against 20% in the treated group (Fig. 5C). In addition, at D4, 1 animal was found dead in the control group. Therefore, bioluminescence quantifications show that the treated group decreased 56% (D2) and 54% (D4) of the total flux compared to that of the control (Fig. 5D). This trend was also observed on the CFU load at D4 (Fig. 5E). After image acquisitions (D4), 2 animals/group were euthanized due to ethical criteria. At the end of the experiment, 7 days after infection (D7), the treated group showed a decrease of 86% of bacterial load compared to that of the control (Fig. 5E). Macroscopic observation revealed that one animal from the control presented several organs with necrosis (liver, spleen, intestine, kidneys, and bladder). Finally, the treated group had an increased survival rate of 50% at D7 compared to that of the control (Fig. 5E).

## DISCUSSION

Bioinspired peptides are increasingly being explored as alternative biofilm controls and have become important allies in the fight against bacterial tolerance and resistance (28, 29). As shown here, capsicumicine, a peptide derived from *Capsicum baccatum* red pepper seeds, possesses strong antibiofilm activity *in vitro* and *in vivo*. We demonstrated that capsicumicine strongly prevents biofilm formation for the most frequent bacteria related to nosocomial infections, S. epidermidis and S. aureus. However, all staphylococci do not produce a biofilm rich in polysaccharides, and considering the proposed mechanism of action of capsicumicine, this might be a limitation to our approach.

**FIG 6:**
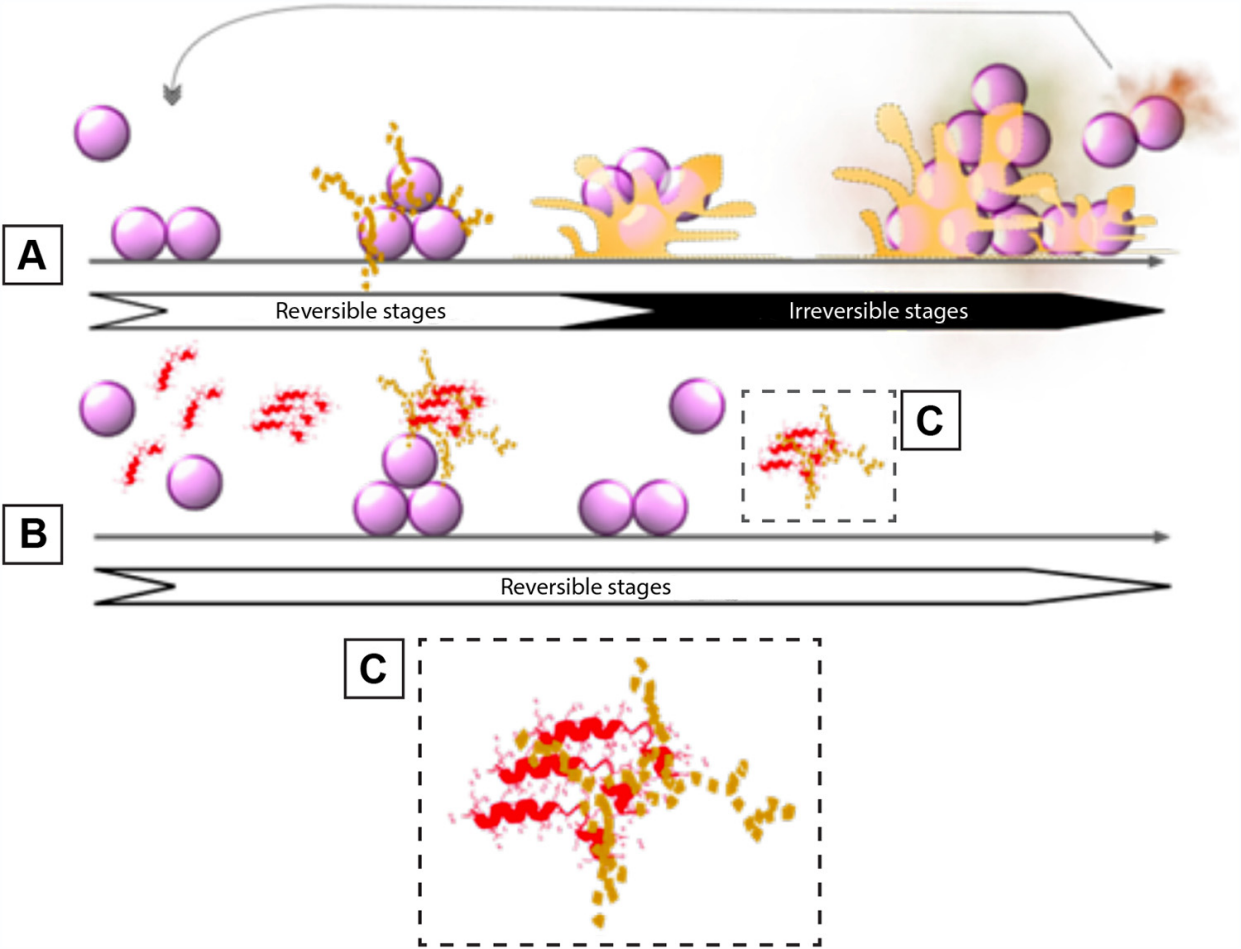
The matrix anti-assembly (MAA) antibiofilm mechanism of action identified in capsicumicine. (A) Untreated biofilm development on abiotic surfaces: planktonic cells interact passively with the surface and start the extracellular matrix production. Initial adhesion is established, after which matrix self-assembly occurs, leading to irreversible biofilm structuring and adhesion. (B) Biofilm development in the presence of capsicumicine shows the MAA mechanism of action: planktonic cells are still able to interact passively with the surface. At that point, capsicumicine acts, attracting extracellular saccharides and shifting the matrix self-assembly. (C) Capsicumicine (red) tends to organize itself in beta leaves favoring the interactions with matrix target-saccharides (yellow). These interactions occur between the noncatalytic carbohydrate-binding modules of the peptide and the saccharides. This peptide supra organization can compete with matrix assembly, preventing/destroying intramatrix H-bonds over a large range of residues. Consequently, the MAA decreases adhesion and aggregation, forcing bacteria to remain planktonic. The arrows indicate reversible (white background) and irreversible stages (black background) during biofilm formation.

Capsicumicine prevents the establishment and maintenance of biofilm architecture through a mechanism of action that we named “matrix anti-assembly” (MAA). MAA differs from matrix disassembly (30), as instead of destructuring preestablished matrices, it acts on the initial phase of assembly preventing its correct structuration. In fact, established biofilms are harder to treat than initial biofilms because they have more complex structures (31), and increased structural complexity means that more energy is required for disassembly (32). Bacterial surface proteins can passively interact with surfaces such as medical devices, generating an initial and reversible adhesion after electrostatic and hydrophobic interactions, Van der Waals forces, and others (33). Bacteria will then require extracellular matrix production in order to remain attached after these weak interactions (34). During this process, physicochemical interactions drive molecular and colloidal matrix self-assembly, establishing a chain of dense architecture that results in stable adhesion (Fig. 6A) (35). In contrast, capsicumicine might interact with matrix saccharides and modify the self-assembly chain, resulting in a less dense and nonfunctional matrix and impaired biofilm formation (Fig. 6B). The capsicumicine amino acid sequence contains several residue characteristics for recognizing putative chitin-binding domains, including polar and hydrophobic residue (45% of Q) and cysteines (36–39). The chitin-binding domain is a well-conserved amino acid stretch that binds specifically to *N*-acetylglucosamine, a homologous structure of PIA (27, 40, 41). Additionally, noncatalytic carbohydrate-binding modules (CBMs) are contiguous amino acid sequences with a discreet fold displaying carbohydrate-binding activity. In this context, capsicumicine displays homologies with all tested CBM proteins, notably with the IcaA protein, which is a PIA synthetase from the same S. epidermidis strain studied here (see Fig. S3 and Table S2 in the supplemental material). According to the CAZY web site, there are currently 86 CBM families defined (http://www.cazy.org/Carbohydrate-Binding-Modules.html). CBMs exhibit different folds, with secondary structures ranging from mainly β-sheet-based organization to a mixture of α-helices and β-sheets, and usually display a high content of loops and unfolded parts. Likewise, the capsicumicine secondary fold is a mixture of α-helix, β-sheet, and unfolded regions, with conformational time and temperature interconversion in favor of β-sheet and unfolded segments. The β-sheet substructure observed in the CD concerns the Nter part of the peptide according to the chemical shifts of the corresponding amino acids (between 8.6 and 9.0). The TOCSY spectra analyses disclose that the conformational changes observed through time originate around the two cysteines, possibly involved in intermolecular disulfide bonds. This disulfide bond is possibly intermolecular since the formation of an intramolecular disulfide bond is only possible by bending the structure to get both cysteines close to each other. The size of different conformers and potential polymers are limited as shown by the NMR peaks of free peptides, which are not broadened at day 7 (Fig. 4C). As a result, these conformational changes may correspond to structural requirements for interactions with the staphylococcal matrix.

The proposed mechanism of action of capsicumicine matrix anti-assembly (MAA) is based on a set of intermolecular and cooperative forces that might be triggered by the peptide to disrupt matrix assembly.

### Intermolecular forces.

The staphylococcal matrix is composed mainly of polysaccharides but also contains proteins (AtlE, Aap, Empb), teichoic acids, and extracellular DNA (42). These saccharides are PIA/PNAG, homoglycans of beta-1,6-linked 2-amino-2-deoxy-d-glucopyranosyl residues. The matrix contains positively charged amino groups (PNAG) as well as negative charges from *O*-succinylation, which confers electrical charge lability on the matrix. Supported by NMR observations, capsicumicine interacts with free chitosan, a PNAG mimetic, and then switches to a higher-molecular-weight organization (Fig. 3B). The line broadening observed with the peptide-chitosan mixture after 1 day evidences the aggregation of chitosan particles with capsicumicine over time as schematized on Fig. 6. Likewise, CSP analysis reveals that chitosan is able to bind preferentially the capsicumicine N terminus (Fig. 4E) before intermolecular disulfide bridge formation and the relative conformational transition. This is supported by the fact that chitosan is substoichiometric at the end of the NMR titration and because the capsicumicine spectrum of the mixture is different from the spectra of the free peptide (Fig. 4C). These interactions may proceed by both hydrogen bonding between the hydrophilic Q lateral chains and hydrophobic/stacking interactions involving I and L residues. According to NMR, the N-terminal part of the peptide is probably the favored interacting region with the carbohydrate moieties. Thus, these data support the MAA model, in which the peptide supra organization would compete with matrix self-assembly preventing intra h-bonds over a large range of residues. However, the NMR results obtained with assembled chitosan, mimicking a preformed biofilm, show that capsicumicine does not interact with the preassembled matrix (Fig. 3C and D), such as demonstrated in the eradication test. Additionally, the amino acids in capsicumicine are mostly neutral, counting 13 Q and 3 S, although due to the Q amide group and S hydroxyl group, they can generate electronegative (dipole-dipole) zones. This high electronegativity suggests that capsicumicine may interact with the positive free charges of the PIA/PNAG. Thus, these polar nonionic forces perform moderate interactions with the polysaccharides. Since strong interactions, such as ionic forces, may trigger unwanted effects including matrix repulsion or sequestration (43, 44), the moderate interactions of capsicumicine seem to be ideal.

### Cooperative polymerization forces.

In living systems, biomolecules perform their functions in the presence of various macromolecules of different shapes and sizes, and these interactions can include cooperative polymerization forces such as depletion forces (DFs) and subsequent molecular crowding (MC) (45, 46). These forces are noncovalent and nonspecific physicochemical interactions, leading to bridging, aggregation, and rheological variations (47, 48), as is observed in the presence of capsicumicine (Fig. 2D to G and Fig. 3A), and also may contribute to MAA. In a suspension containing different molecules, DFs are the pressures exerted by small particles, which in turn cause attractive forces between the macromolecules. DFs are only expressed in crowded environments like biofilms, driving the assembly and final shape of these structures (49). Taking together NMR and CD experimental results as well as the TANGO and SALSA analysis (see Fig. S5B in the supplemental material), we can picture that capsicumicine can self-organize to form extended structures both by disulfide bridges and β-sheet-mediated fibril formation. We propose that capsicumicine-associated DFs may coagulate in polymer solutions forming fibers and parallel bundles (50), explaining the observed branch-like profile (Fig. 2D to G) (51). This higher organization would facilitate the interaction with the saccharides of the matrix preventing their pattern assembly and resulting in flocculation (52). In the same way, MC in macromolecule solutions is characterized by a decrease in accessible volume due to high macromolecule concentrations, as well as attractive and repulsive forces between them (45, 53). Some molecular crowders modulate refolding kinetics and decrease competing aggregation and segregation (54). In this way, capsicumicine might enhance the aggregation kinetics (Fig. 3A), acting as an agent of MC, decreasing entropic forces, and leading to segregation (55). The *in vitro* interactions between capsicumicine and chitosan demonstrate that capsicumicine’ structures are chemically able to interact with staphylococcal matrix saccharides. However, natural staphylococcal matrices are rather rich in PNAG and of course far more complex, making interactions with other matrix constituents also possible. These combined intermolecular and cooperative forces might disturb matrix self-assembly at both molecular and colloidal levels. Consequently, matrix functionality would shift and antibiofilm activity occur. Additionally, planktonic microorganisms are more available for the innate immune system to recognize and clear (56). Furthermore, nonantibiotic activity appears less susceptible to the development of bacteria resistance because these microorganisms are under less evolutionary pressure then when exposed to conventional antibiotics (57, 58).

Finally, although there are no antibiofilm drugs available yet, here, we relate the discovery of a new carbohydrate-binding peptide as a promising candidate for complementary drug/treatment of infectious diseases. In particular, we propose its antibiofilm mechanism of action, matrix anti-assembly (MAA), and validated a proof of concept for an *in vivo* application.

## MATERIALS AND METHODS

### Peptides.

All peptides were synthesized by Biomatik and ProteoGenix at purity grades over 95% in salts suitable for cell culture. For the assays, the peptides were all solubilized in ultrapure sterile water.

### Bacterial strains and growth conditions.

Staphylococcus epidermidis ATCC 35984 was grown overnight on blood agar (Thermo Scientific Oxoid; PB5039A) at 37°C. Oxoid LB agar was used for the CFU assay. The other assays were done using a bacterial suspension of 3 × 10^8^ CFU/ml in tryptone soya broth (TSB) (Oxoid) or 0.9% NaCl.

### Biofilm formation.

At least three technical and biological replicates were done for each assay of 1, 10, or 100 μM peptide concentrations.

**(i) Biofilm formation inhibition.** A protocol adapted from Trentin et al. (59) was used with crystal violet (CV) in 96-well BD Falcon polyvinyl chloride (PVC) microtiter plates. The cell-bound stains were solubilized with absolute ethanol (Sigma-Aldrich), and absorbance was measured at 570 nm using a BioTek PowerWave XS plate reader. The biofilm formation control represents 100% of biofilm formation.

**(ii) Biofilm eradication.** Biofilm was preformed as described above for 24 h at 37°C without treatment. Afterwards, the wells were washed to remove planktonic cells, peptide solutions and controls were added, and all were incubated for 24 h. Biofilm eradication was verified by CV as previously described.

### Bacterial growth assays.

**(i) Microtiter plates.** Bacterial growth was evaluated by comparing OD_600_ values at the start and end of incubation in 96-well PVC microtiter plates. After incubation at 37°C for 24 h, CFU per milliliter was calculated to determine the peptide solution’s bactericidal effects. The untreated bacteria were considered as the growth control. At least three technical and biological replicates were performed for all assays.

**(ii) Quantitative reverse transcription-PCR.** After culturing for 24 h, RNAs were isolated from planktonic controls, biofilm controls, and from total cells exposed to 10 μM capsicumicine. An Invitrogen TRIzol Max bacterial RNA isolation kit and an Ambion TURBO DNase treatment were used as per manufacturer instructions. Total RNA concentrations and purities were assessed using a Biochrom SimpliNano spectrophotometer, and PCRs were performed to ensure the complete absence of DNA. Each qRT-PCR was then subjected to previously established quantities of cDNA (10 ng) and primers (0.2 μM). Reactional volumes and conditions were used as per the manufacturer’s instructions (SYBR Select master mix; Applied Biosystems). Primers (see Table S1 in the supplemental material) were designed using the Primer3 program and then produced by Eurofins Genomics. Applied Biosystems StepOnePlus equipment and software were used. Relative transcript levels were determined by the 2^−ΔΔct^ method (60).

**(iii) Cytotoxicity assays.** Cytotoxicity assays were performed on the ImPACcell robotic platform (BIOSIT, Université de Rennes 1). Multiparameter high-content screening (HCS) and high-content analysis (HCA) of chemical markers related to cell viability were done on 7 different mammalian lines as follows: HuH7 (hepatocellular carcinoma), Caco-2 (colorectal adenocarcinoma), MDA-MB231 (breast adenocarcinoma), HCT116 (colorectal carcinoma), PC3 (prostatic adenocarcinoma), NCL-H727 (lung carcinoma), and MCF7 (breast cancer). The number of viable cells is presented as residual cell percentage compared to the dimethyl sulfoxide (DMSO) control average.

### Microscopic analysis.

S. epidermidis ATCC 35984 biofilm was cultured as described above.

**(i) Scanning electron microscopy.** Sterile 10- by 4-mm polystyrene coupons were inserted into bacterial cultures in the presence or absence of capsicumicine for 1, 4, and 24 h. The coupons were then washed with sterile 0.9% NaCl and fixed with 2.5% glutaraldehyde, 2% paraformaldehyde, and 0.1 M cacodylate buffer (pH 7.2). Afterward, they were washed with 0.1 M cacodylate buffer and 0.2 M sucrose and then dehydrated with increasing concentrations of ethanol. A Leica EM CPD300 was used for critical point drying of the dehydrated samples. These were then sputtered with palladium in a Leica EM ACE200 and analyzed with a JEOL JSM-7100F microscope with energy-dispersive X-ray spectrometry (EDS) and electron backscatter diffraction (EBSD) at 10 kV.

**(ii) Transmission electron microscopy.** All well content was carefully detached at 1, 4, and 24 h, centrifuged at 10,000 × *g* for 15 min at 4°C, and then washed with sterile 0.9% NaCl. Fixation was performed at 4°C with sodium 0.1 M cacodylate, 2% paraformaldehyde, 2.5% glutaraldehyde, and 75 mM lysine. Samples were washed with 0.1 M sodium cacodylate and 0.2 M sucrose and contrasted with 1% osmium tetroxide and 1.5% potassium ferrocyanide. Dehydration was done with a gradual solution of ethanol and infiltration of increasing concentrations of LR white resin (Delta Microscopies, France). LR white resin inclusion and polymerization were then performed over 24 h at 60°C in the absence of O_2_. Thin 80-nm sections were collected onto carbon grids and visualized at 200 kV with an FEI Tecnai Sphera microscope equipped with a Gatan 4k x 4k charge-coupled device (CCD) UltraScan camera.

**(iii) Confocal fluorescence microscopy.** Capsicumicine-fluorescein isothiocyanate (capsicumicine-ITC) (10 μM) was used to detect the capsicumicine peptide, whose antibiofilm activity was previously verified. After incubation for 1, 4, or 24 h, the well contents were carefully detached, centrifuged at 11,000 × *g* for 2 min at 4°C, and then washed with sterile 0.9% NaCl. The suspension was visualized directly or after adding 0.1 μg/μl concanavalin A conjugates (Alexa Fluor 633; Invitrogen) or 2 mg/ml Calcofluor white dye (Fluorescent Brightener 28; Sigma-Aldrich). The pseudonajide FITC-labeled peptide is a known antimicrobial peptide that permeates the bacterial cell and was used as a permeability reference (61). Images were acquired via resonant scanner with a Leica SP8 DMI 6000 CS confocal microscope with hybrid detector, and ImageJ software was used for image analysis.

### Real-time molecular self-assembly assay.

The assembly reaction of the synthetic staphylococcal matrix, starting at pH 7.2, was recorded by measuring the OD_600_ as a function of time every 30 s for 30 min (35). Molecular self-assembly reactions were calculated to a final volume of 4 ml, with 0.3% chitosan (medium molecular weight, 75 to 85% deacetylation), 0.15% bovine serum albumin, and 0.015% lambda DNA (all from Sigma) in TSB. The concentration (μM) of tested peptides was calculated for a final volume of 4 ml. Before getting the assembly reaction pH starting points, a calibration record was done using the same reactional tube containing all reagents (auto zero). Acetic acid and NaOH were used to adjust pH, and the reaction temperature was about 30°C. As a negative control, a similarly sized peptide was used, PA-1 (62).

### NMR.

NMR spectra were recorded in 3-mm tubes on a Bruker Avance III 600-MHz spectrometer equipped with a TXI (1H,13C,15N) probe and a Z-gradient unit. Spectra were processed with TopSpin 4.0.8 (Bruker Biospin) and CcpNmr analysis (63). TOCSY and NOESY experiments were respectively recorded at 10°C and pH 5.0 with a 70 ms and 300 ms mixing time. Capsicumicine concentration in water was set at 0.3 mM and the pH adjusted either to 5.0 or to 3.5 with a few microliters of deuterated HCl 0.1 M and/or NaOH 0.1 M solutions. Chitosan powder (purchased from Sigma) was dissolved in pure water and the pH adjusted to either 5.0 or 3.5. Reference 1D proton NMR spectra were recorded at 10°C and either at pH 3.5 and 5.0. Chitosan was stepwise added from a concentrated solution at pH 5.0 or 3.5, and spectra changes were monitored. We first titrated capsicumicine solution with chitosan solution at pH 5.0. The concentration variation of chitosan was estimated using the peaks intensities, resulting in 3.6 and 3.8 ppm (not shown) in the beginning to less than 0.5 eq at the end of the titration. These experiments were repeated with a starting capsicumicine solution at pH 3.5 and the gradual addition of gelled chitosan at pH 3.5 and the sudden addition of gelled chitosan pellets in the NMR solution at pH 3.5.

### Circular dichroism.

The peptide was dissolved in 18 M Ωwater at a concentration of 50 μM at pH 5.0. The UV CD spectra were recorded at 5, 15, and 30°C in a 0.1-cm path length quartz cell on a Jobin Yvon CD6 spectrometer equipped with a temperature controller unit over a 180- to 260-nm range with a 2-nm bandwidth, a step size of 1 nm, and an integration time of 2 s per point. The samples were conserved at 5°C between each recording. Spectra were averaged over 5 records. Water CD contributions were subtracted from CD spectra before processing. Spectra were processed using KaleidaGraph (Synergy Software). Molar circular dichroism (Δε) per residue and molar ellipticity per residue (θ) (MER) were computed from the difference of the delta absorbance recorded by the spectrometer. Raw delta epsilon per residue spectra were analyzed using the CDSSTR program (64) and different reference protein data sets provided by the DICHROWEB facility (65). MER data curves were smoothed for presentation, using the interpolate and weighted data (5%) routines provide by KaleidaGraph.

### TANGO and SALSA predictions.

Tango (66) and Salsa (67) predictions were run using the AMYpdb online web server (http://amypdb.genouest.org/e107_plugins/amypdb_project/project.php) (68). For Tango temperature was set to 298 K. Predictions were both run with pH set either to 5.0, 7.0, or 9.0. SALSA predictions were run with a window size dynamic 512 of 4 to 20 residues, a cutoff of 1.2, and a minimal hot spot length of 5.

### CVC coatings.

We adapted an approach to immobilize peptides based on poly(ethylene glycol)diacrylate (PEGDA) hydrogel (69). Briefly, polyurethane (PU) tubes (Instech) of 2 Fr or 25 G equivalent (0.4 to 0.7 mm of diameter) were used as coating framework. Hydrogel base was prepared using pentaerythritol tetrakis(3-mercaptopropionate) (PTMP) (4.1 mmol), PEGDA (10 mmol), PEG-600 (20 mM), 2,2-dimethoxy-2-phenylacetophenone (DMPA) (0.1 wt%), THF soluble PU (10 wt%), and methanol (qs). Peptides were solubilized in DMSO and added to a hydrogel base under vortex agitation. PU tubes were first internally coated by suction using a needle syringe and then externally by immersion. The reaction and polymerization conditions such as room temperature and oxygen tolerance were easily implemented. After polymerization, successive washes with methanol under agitation were used to eliminate undesirable monomers. All reagents were purchased from Sigma-Aldrich.

### CVC infection assay.

This study was conducted by Voxcan s.a.r.l. The ethical agreement is part of project APAFiS number 10756-2017072522272676 v4, approved by the Voxcan ethical committee (CEAA-129) and the French authorities (Ministry of National Education, the Higher Education and Research). This study used S. aureus rather than S. epidermidis for the *in vivo* experiment because of the poor bioluminescent activity of the known S. epidermidis strain. Indeed, the previously described bioluminescent S. epidermidis strain is no longer available, as its light-emission levels are not stable (see Fig. S1 in reference 70). Since the use of bioluminescence for quantifying bacterial colonization and biofilm formation significantly reduces the number of animals needed for testing, thus decreasing the need for euthanasia at each point/time in the analysis, we decided to base the study on bioluminescent S. aureus. We therefore first validated the *in vitro* activity of capsicumicine with the bioluminescent S. aureus (see Fig. 5b). This study used SKH1 mice, females, immunocompetent, and specific pathogen free (SPF) provided by Charles River Laboratories. Animals were acclimated at least 2 days, housed collectively in disposable standard cages in ventilated racks A3 biological safety, at +21 ± 3°C, 30 to 70% humidity, and 12-h dark and light cycles, with filtered water and autoclaved standard food provided *ad libitum*. Catheters were blind implanted (*n* = 5/group) in mice jugular vein followed by an intravenous (i.v.) inoculation of S. aureus Xen36 5 days later, which was in turn to colonize the device from blood circulation. The bacterial development was evaluated and compared between the different catheters by *in vivo* bioluminescence imaging (IVIS Spectrum; acquisition and analysis with Living Image version 4.5.5) performed 2 and 4 days after mice infection. In addition, 7 days after inoculation or at the time of mice euthanasia for ethical reason, catheters were harvested and bacterial load was evaluated by CFU counting (SCAN500). All along the study, mice clinical state was evaluated using a scoring grid and body weight measurements 3 times a week. At each time point, a bioluminescence acquisition was also performed on the background (BKG) mouse to measure the flux level corresponding to the autobioluminescence. Images were radiance-thresholded with respect to the background radiance level and smoothed with a bidimensional Gaussian filter (3 by 3).

### Data availability.

All data needed to evaluate the conclusions in this article are present in the paper and/or the Supplemental Material. Additional data related to this paper may be requested from the authors.
