# Supplementary material for: Capsicumicine, a New Bioinspired Peptide from Red Peppers Prevents Staphylococcal Biofilm In Vitro and In Vivo via a Matrix Anti-Assembly Mechanism of Action
Source: Microbiol Spectr. 2021 Oct 27;9(2):e00471-21. doi: 10.1128/Spectrum.00471-21 (PMC8549733; doi:10.1128/Spectrum.00471-21)
Supplement: SUPPLEMENTAL FILE 1 — Supplemental material. Download SPECTRUM00471-21_Supp_1_seq4.pdf, PDF file, 2.5 MB [file spectrum00471-21_supp_1_seq4.pdf]

Supplementary Materials for

Capsicumine, a new peptide bioinspired from red peppers, prevents staphylococcal biofilm *in vitro* and *in vivo via* a matrix anti-assembly mechanism of action

Rafael Gomes Von Borowski[1,2], Sophie Chat[1], Rafael Schneider[1,2], Sylvie Nonin-Lecomte[3], Serge Bouaziz[3], Emmanuel Giudice[1], Aline Rigon Zimmer[2], Simone Cristina Baggio Gnoatto[2], Alexandre José Macedo[2,4,*], and Reynald Gillet[1,*]

[1] Univ. Rennes, CNRS, Institut de Génétique et de Développement de Rennes (IGDR), UMR6290, Rennes, France; [2]Programa de Pós-Graduação em Ciências Farmacêuticas, Faculdade de Farmácia, Universidade Federal do Rio Grande do Sul, 90610-000 Porto Alegre, Brazil; [3]Université de Paris, CNRS, CiTCoM (Cibles Thérapeutiques et Conception de Médicaments) UMR 8038, Faculté de Pharmacie, 75006 Paris, France; [4]Centro de Biotecnologia da Universidade Federal do Rio Grande do Sul, 91501-970 Porto Alegre, Brazil

[*]To whom correspondence may be addressed. Email reynald.gillet@univ-rennes1.fr or alexandre.jose.macedo@gmail.com.

Table S1. Primers used in this study. These were previously designed using the Primer3 program and standardized by our team.

Gene	Forward primer 5'-3'	Reverse primer 5'-3'	Amplicon
atlE	TACCAGGGTTTGCAGGATTC	GGCGCTAAATTCATTGGAAA	85 pb
aap	AGGCCGTACCAACAGTGAAT	ATGGGCAAACGTAGACAAGG	100 pb
agrC	TCATCAATATCGCATTCATCG	CCTAAACCGCGATTATCACC	136 pb
icaA	TTATCAATGCCGCAGTTGTC	CCGTTGGATATTGCCTCTGT	104 pb
leuA	GATGATCTCGGAATGGCAGT	TGAGGCATTTCCTGCTCTTT	108 pb
saeR	GCTAACACTGTCAATGTCCACA	AGGCCCCACACAGTTGTAAT	92 pb
saeS	GGCGTCAATTTGTTGTGCTA	AGGGCATAGGTATCGTTCCA	140 pb
sarA	TTTGCTTCTGTGATACGGTTGT	CGTAATGAACACGATGAAAGAACT	107 pb
gyrB	ATCAACATCGGCATCAGTCA	GCATTTGGTACGGGTATTGG	87 pb
rrsA	AAGCAACGCGAAGAACCTTA	ATGCACCACCTGTCACTCTG	95 pb

Table S2. Carbohydrate-binding domain proteins that are capsicumicine homologs. These proteins are available in the UniProtKB database and were used for BLAST and amino acid alignments to perform similarity analysis.

Entry	Name	Accession ID
ICAA_STAEQ	Intercellular adhesion protein icaA	Q5HKQ0.1
ICAD_STAEQ	Poly-beta-1,6-N-acetyl-D-glucos. synthase prot. icaD	Q5HKP9
A7Z8H9_BACVZ	Chitosanase	A7Z8H9
A0A0N0MLT5_9ACTN	Chitosanase	A0A0N0MLT5
CBP2_MOROL	Chitin-binding protein 2	C0HKC5
A0A1R0GTZ5_9FUNG	Chitin synthase 8	A0A1R0GTZ5
A0A194V113_9PEZI	Chitin biosynthesis protein CHS5	A0A194V113

Table S3. Results of the CD spectra deconvolution using CDSSTR algorithm.

Day	Temp (°C)	Helix (%)	Bêta (%)	Turn (%)	Unfolded (%)
0	5	42-46	29-34	5-8	16-21
1	5	42-45	30-32	6-8	20-21
5	5	38-42	24-31	8-12	17-23
5	15	22-26	36-45	13-14	20-26
5	30	16-24	33-40	18-20	20-25
7	30	6-17	38-41	10-23	31-35

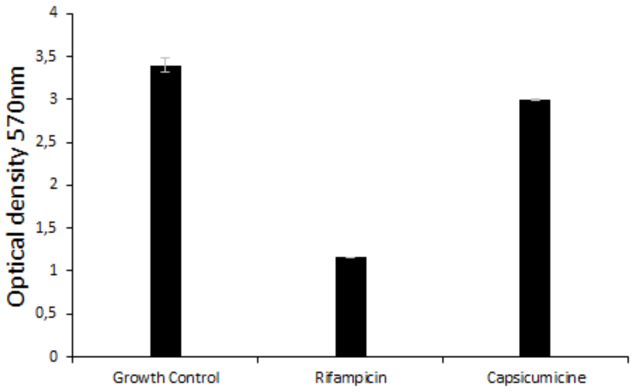

Fig. S1. Biofilm eradication test. Shown are *Staphylococcus epidermidis* (ATCC 35984) biofilm quantifications at OD_{570} for the bacterial biofilm without peptide exposure (“Growth Control”), after exposure to the rifampicin antibiotic control, and after 24 h treatment with 100 µM capsicumicine.

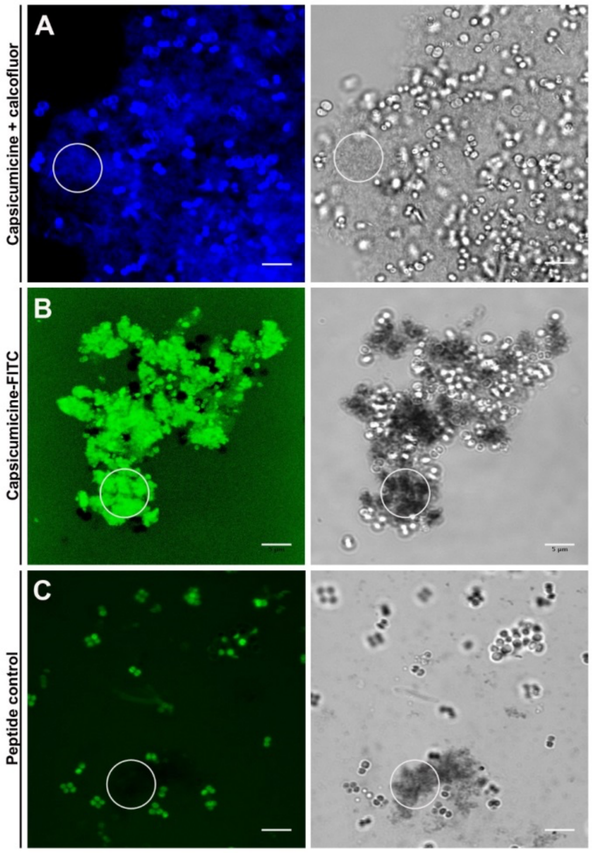

Fig. S2. Confocal fluorescence microscopy (CFM) of *Staphylococcus epidermidis* (ATCC 35984). Calcofluor was used to highlight matrix polysaccharides (blue), and FITC used for the peptides (green). Visualization was done by fluorescence (left) and transmitted light (right) microscopy. (*A*) Cultures exposed to capsicumicine and calcofluor. (*B*) Cultures exposed to capsicumicine-FITC. (*C*) Peptide negative control cultures exposed to an antimicrobial peptide-FITC (Pseudonajide). The matrix is highlighted (circles). Scale bars, 5 µm.

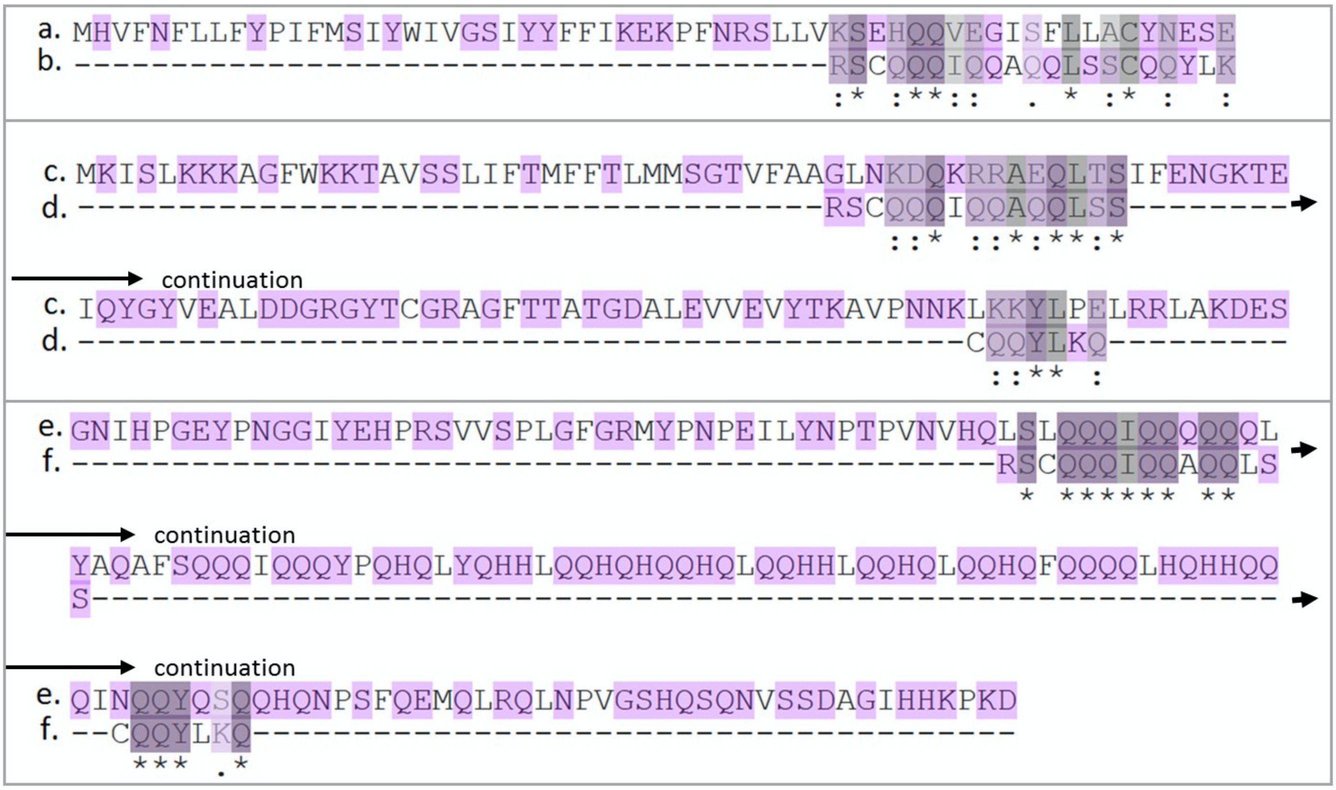

Fig. S3. Amino acid alignment of capsicumicine and its carbohydrate-binding domain protein homologs. (*a*) The icaA (protein accession number Q5HKQ0.1) fragment from 1 to 60. (*b*) Capsicumicine fragment from 1 to 21. (*c*) Chitosanase (A7Z8H9) fragment from 1 to 60 and from 61 to 120. (*d*) Capsicumicine fragment from 1 to 15 and from 16 to 22. (*e*) Chitin synthase 8 (A0A1R0GTZ5) fragment from 1801 to 1860 and from 1921 to 1967. (*f*) Capsicumicine fragment from 1 to 14 and from 16 to 22. Equal (* and grey highlighting), similar (.), and highly similar (:) amino acids are indicated, as well as amino acid polar characteristics (purple). Support for this analysis is available at UniProt.

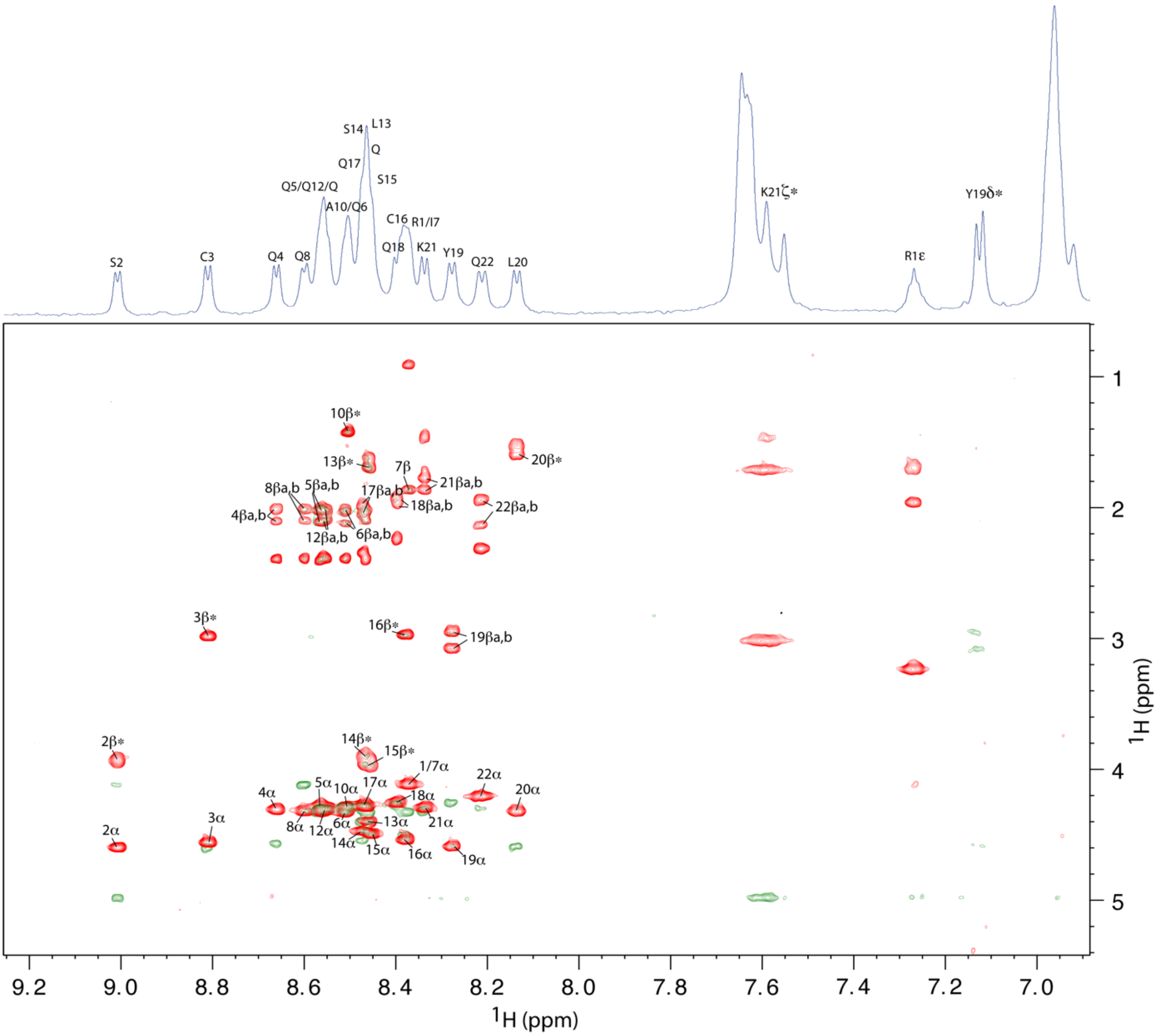

Fig. S4. Superposition of the TOCSY (red), NOESY (green) and 1D (blue) spectra of capsicumicine recorded at pH 5.0 and 10°C. All amide NH resonances were assigned using nNH-(n-1)Hα NOESY cross-peaks, except for Q9 and Q11 residues due to poor resolution. For clarity, only NH-Hα and NH-Hβ labels were reported. The specta display 22 spin systems as expected for a single conformation. According to the spectral dispersion, capsicumicine is mainly folded.

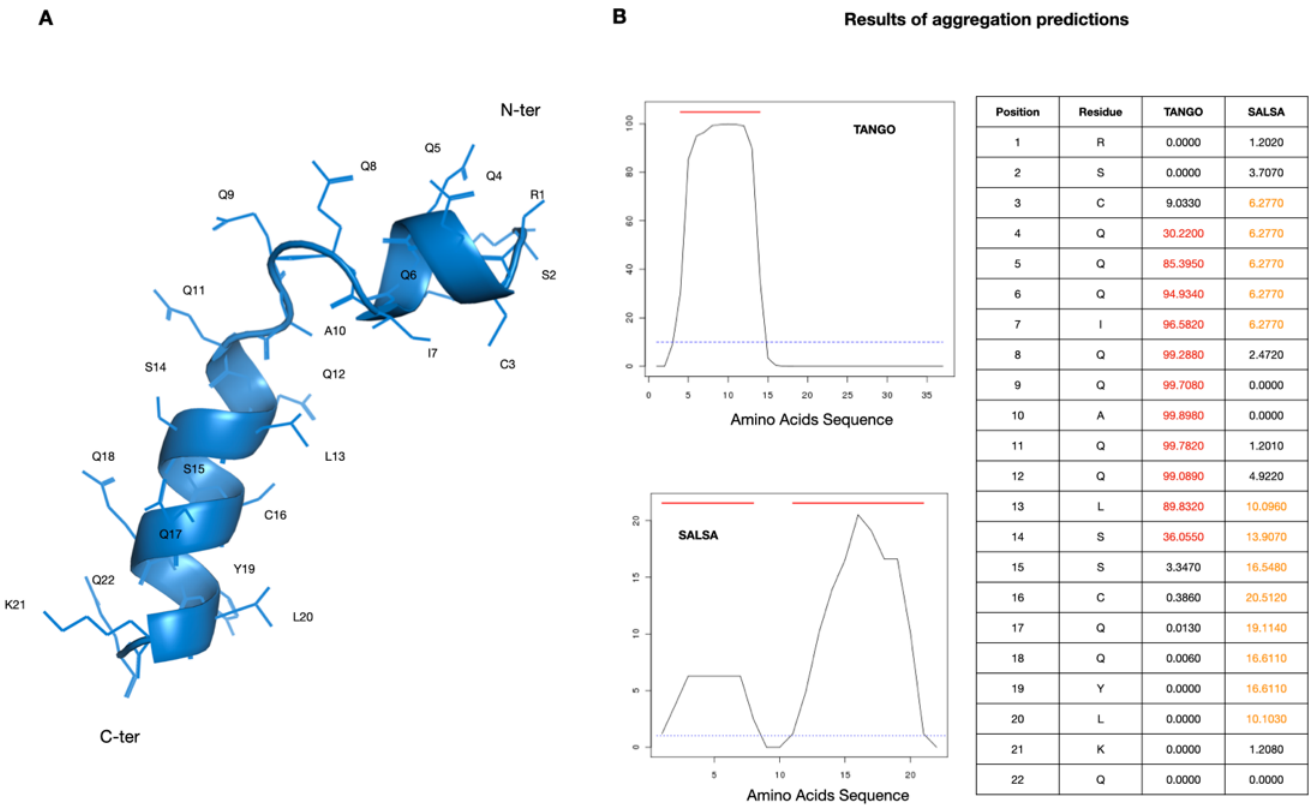

Position	Residue	TANGO	SALSA
1	R	0.0000	1.2020
2	S	0.0000	3.7070
3	C	9.0330	6.2770
4	Q	30.2200	6.2770
5	Q	85.3950	6.2770
6	Q	94.9340	6.2770
7	I	96.5820	6.2770
8	Q	99.2880	2.4720
9	Q	99.7080	0.0000
10	A	99.8980	0.0000
11	Q	99.7820	1.2010
12	Q	99.0890	4.9220
13	L	89.8320	10.0960
14	S	36.0550	13.9070
15	S	3.3470	16.5480
16	C	0.3860	20.5120
17	Q	0.0130	19.1140
18	Q	0.0060	16.6110
19	Y	0.0000	16.6110
20	L	0.0000	10.1030
21	K	0.0000	1.2080
22	Q	0.0000	0.0000

Fig. S5. (***A***) Predicted capsicumicine structure using the Phyre^2 prediction server. (***B***) Aggregation structure predictions using TANGO (1) and SALSA (2) algorithms.

Supplementary References

1. L. A. Kelley, S. Mezulis, C. M. Yates, M. N. Wass, M. J. Sternberg, The Phyre2 web portal for protein modeling, prediction and analysis. Nat Protoc 10, 845-858 (2015).

2. S. Zibaee, O. S. Makin, M. Goedert, L. C. Serpell, A simple algorithm locates beta-strands in the amyloid fibril core of alpha-synuclein, Abeta, and tau using the amino acid sequence alone. Protein Sci 16, 906-918 (2007).
